# A qualitative study exploring experiences and challenges of combining clinical academic training with family life

**DOI:** 10.1186/s12909-021-02849-8

**Published:** 2021-08-16

**Authors:** Diane Trusson, Emma Rowley

**Affiliations:** University of Nottingham, School of Medicine, NIHR Applied Research Collaboration East Midlands (ARC EM), Institute of Mental Health, Triumph Road, Nottingham, NG7 2TU UK

**Keywords:** Clinical academic careers, Nurses, Midwives, Allied health professionals, Medical clinical academics, Family life, Caring responsibilities

## Abstract

**Background:**

Concerns are being expressed around the lack of diversity at higher levels of clinical academia. This study aimed to explore experiences and challenges associated with combining clinical academic careers with family life.

**Methods:**

Qualitative data were gathered from participants from 4 NHS Trusts and 2 universities in the East Midlands of England using online surveys and semi-structured interviews.

**Results:**

The survey was completed by 67 nurses, midwives and allied health professionals, and 73 medical clinical academic trainees. Interviews were conducted with 16 participants from each group including equal numbers of men and women. Caring responsibilities differed between the two study populations. Medical clinical academic trainees were younger and either had young children or were yet to start a family. In contrast, nurses, midwives and allied health professionals tended to be older when they embarked on a clinical academic career and often waited until their children were school-age or older. Similar concerns were raised regarding working part-time and childcare, and how their career prospects might be affected in terms of fulfilling promotion criteria and being able to relocate for work purposes. The occupation of their partners also featured in participants’ experiences; those who shared childcare with someone who worked ‘regular’ hours, appeared to be better supported to combine a clinical academic career with family life.

Gender stereotyping was identified in some reported experiences highlighting a need for appropriate mentorship and for positive role models who were able to demonstrate that it is possible to survive and thrive as a clinical academic with family responsibilities.

**Conclusions:**

Although people manage to find ways to successfully combine clinical academic roles with family life, findings highlight a need to identify ways of supporting and encouraging trainees with caring responsibilities to ensure that they remain on the clinical academic pathway.

## Background

Clinical academics are doctors, dentists, nurses, midwives and allied health professionals who combine clinical practice with academic research and/or teaching. The benefits of undertaking clinical academic training include enhanced career satisfaction and improved prospects for promotion [1]. Clinical academics bring their clinical expertise and experience to research projects which have potentially wide benefits for the health service and patient care [2]. However, there are differences in the clinical academic training offered to doctors and dentists (medical clinical academics, henceforth MCAs) and the academic training provision for nurses, midwives and allied health professionals (henceforth NMAHPs). These differences are discussed fully elsewhere [3] but briefly, MCAs are able to begin clinical academic training from medical school onwards. The National Institute for Health Research funds an integrative clinical academic pathway [4], meaning that MCAs usually undertake academic training in parallel to their medical training. For example, predoctoral Academic Clinical Fellows (ACFs) have 25% of their time dedicated to academic training, and Clinical Lecturers (CLs) split their time 50/50 between clinical and academic commitments [4]. Consequently, MCAs take longer to qualify medically compared to their colleagues who follow a purely clinical route. In contrast, NMAHPs can only begin clinical academic training once they are clinically qualified. Furthermore, unlike their medical colleagues, there is no integrated pathway for NMAHPs which means that they often have to reduce their clinical hours or leave their clinical post, in order to pursue a clinical academic career [3].

Challenges associated with clinical academic training include balancing clinical and academic responsibilities [5], negotiating competing pressures from universities and NHS Trusts, financial concerns, and competition for funding and jobs [3]. Another area of concern for clinical academic trainees is achieving an acceptable work/life balance [6]. Studies of factors influencing trainees’ career decision-making reveal that female students in particular want a career that fits their domestic circumstances and work arrangements such as flexible hours and part-time posts, that are compatible with caring responsibilities [7, 8]. This is becoming increasingly pertinent amid concerns about disproportionately low numbers of female professors and associate professors both nationally and internationally [9] with few people working part-time in these posts [10].

The data discussed in this paper come from a larger study which was the first to compare the benefits and challenges associated with the clinical academic pathway as experienced by NMAHPs and MCAs [3]. Although it was not the main focus of the larger study the topic of family considerations and caring responsibilities was identified as a major theme within participants’ reflections on their career decision-making. It was considered important to pursue this topic in the context of gender disparities at high levels of clinical academia [9], and previous research suggesting that balancing the ‘substantial workload of academic medicine with…childbearing and child rearing’ could prevent postdoctoral trainees pursuing a clinical academic pathway [5].^,p.3^

In contrast to previous studies which often focus on medical academics at particular points in their training trajectory [5–8], this study compares experiences and challenges associated with combining clinical academic careers with family life between MCAs and NMAHPs at various stages of their clinical academic training. Furthermore, the inclusion of male and female participants contrasts with studies which generally focus on women’s perspectives of negotiating work and family [11]. This will allow consideration of the ways in which traditional gender roles are challenged and perpetuated by men and women who are undertaking clinical academic training [12].

The aim is to identify ways in which trainees might be supported to pursue a clinical academic career alongside family responsibilities and avoid attrition of talented individuals.

## Methods

**Fig. 1:**
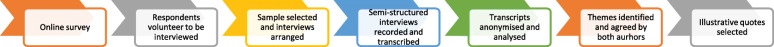
Study design

This study used qualitative methods to explore individual experiences of clinical academics from a range of healthcare professions and at various stages of training [13]. The studies of NMAHPs and MCAs were conducted separately, but each used similar methodology as shown in Fig. 1.

A link to an online survey was sent to members of a clinical academic network for NMAHPs and also to a database of 263 medical clinical academic trainees in the East Midlands of England. The survey respondents were invited to be interviewed, out of which 16 NMAHPs and 16 MCAs were selected. This number allowed a range of data to be collected within a reasonable time frame whilst ensuring that equal numbers of men and women from a variety of occupations and training stages were represented within each group for comparative purposes. This number was also considered sufficient to reach data saturation [13].

Interviews were conducted by the lead author, a female Research Fellow with a PhD in sociology and extensive experience of conducting social research but no medical background or established relationship with the research participants. The same set of questions was used for both sets of participants, except for an additional question in the MCA study relating to combining clinical academic careers and family life. This was prompted by the rich unsolicited data on the topic that arose in the NMAHP’s study which was felt worthy of pursuit when comparing the two groups [3]. The interviews which lasted between 30 and 60 min were digitally recorded with permission, and professionally transcribed. All identifying details were removed from the data prior to analysis.

**Fig. 2:**
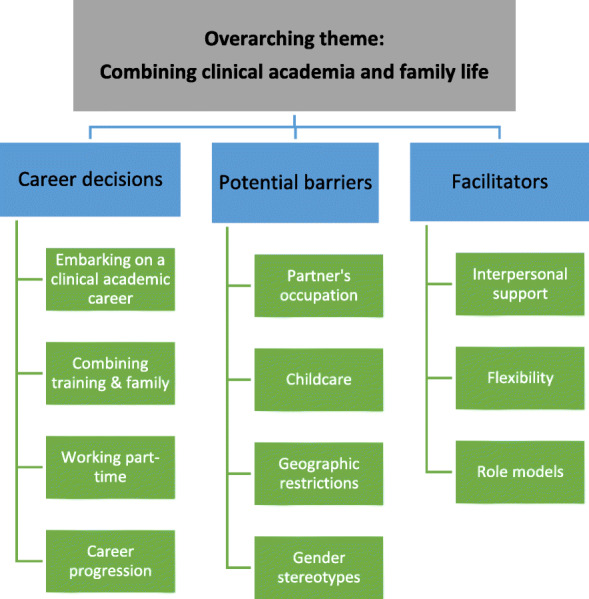
Coding tree

The qualitative data from responses to an open survey question around anticipated challenges in pursuing a clinical academic career were combined with the interview data and analysed using a framework analysis [14]. This method is not aligned with a particular theoretical approach but allows themes to emerge through the data using an inductive process [14]. The lead author familiarised herself with the data through multiple readings and identified several themes and sub-themes which were discussed and agreed with the co-authors (see [3]). The theme of combining clinical academic training with family responsibilities (as shown in Fig. 2) is the focus of this paper.

### Ethical considerations

Following Health Research Authority [15] guidance, this research was not submitted for Ethics Board approval because it was considered to be an evaluation of a training programme; also participants were recruited by virtue of their participation in educational programmes, rather than their NHS status [15]. Nevertheless, the study was conducted in accordance with the Declaration of Helsinki [16]. All the data excerpts included in the results section are identified by the survey respondent or interviewee participant number, along with their self-identified gender and training stage (MCA) or clinical role (NMAHP).

## Results

The survey was completed by 67 NMAHPs (11 men, 55 women, and one participant who preferred not to say) and 73 MCAs (41 men and 32 women). The majority of NMAHPs (37.3%) were aged between 31 and 40, with equal numbers (25.4%) aged between 20 and 30 or 41+. In contrast, most MCAs (65.8%) were aged between 20 and 30; with 32.9% aged between 31 and 40 and one aged 41+. This had consequences for participants’ caring responsibilities in that the comparatively older NMAHP respondents were more likely to have children. Although they were not explicitly asked about their families, interviews with NMAHPs revealed that one participant was expecting her first child, 6 discussed children who were either school age or older, and 2 participants (both male) said they had young children. However, it is also possible that some may have not mentioned their child(ren). Reflecting on a study of NMAHP pre-doctoral students (mainly women, aged between 25 and 55) [17], one participant noted that students with children rarely raised the topic of being parents, whereas those without children wondered how people managed to combine their clinical academic training with caring responsibilities.

### Embarking on a clinical academic career

Some participants described their family as providing motivation for pursuing a clinical academic career:

I’m very fortunate; my wife really supports me, so I don’t want to let her down. We have two young kids, and she knows this has been an ambition of mine for around nine years now…to do this would make her very proud; to pull off the research, write the thesis and do it; to be able to tell the kids about it I suppose (Male Interviewee 16, Allied Health Professional,).

Another male participant described how he found it difficult to work on his PhD until the early hours when he had a young baby to care for but considered it to be worth the effort at this stage in his life. Both these participants were unusual within the NMAHP study in pursuing a clinical academic career while their children were young, but they implied that they were making sacrifices now for their future careers. Conversely, many (female) NMAHP respondents described waiting until their child(ren) reached school age.

However, this was not considered the best approach by one participant who wanted to encourage NMAHPs to pursue a clinical academic career earlier:

We need newly qualified midwives as well as those who’ve been around for years. I was one of them, waiting for their families to get to a point where they could put a bit more effort into their career, there’s plenty of those! (Female Interviewee 4, Midwife).

In contrast, MCAs were younger, having embarked on academic training in tandem with their clinical training. Responses to the MCA survey question about anticipating challenges in pursuing a clinical academic career included taking time out for maternity/parental leave and/or working part-time in the future; these were mentioned by more female [11] than male [5] respondents.

### Combining clinical academic training and family life

Nine of the MCA interviewees (2 men and 7 women) described having young children; 3 of them (all female) worked part-time. Some (female) participants raised the topic of family responsibilities unprompted, early in their interviews when describing their experiences of being a clinical academic. When asked for their perspectives on combining clinical academic careers and family life, most participants discussed issues around childcare, part-time working, and the impact of these issues on their career aspirations.

While it is well-documented that balancing clinical and academic commitments can be challenging [1, 3, 5], participants with family caring responsibilities expressed additional concerns:

At times, it’s been really hard…Because you’re splitting your time so thinly on all these different projects...and then obviously throw motherhood into that as well, never a good enough mother or doctor or academic, but at the same time, it keeps me interested (Female Interviewee 12, Clinical Lecturer).

These comments illustrate the push and pull of pursuing a clinical academic career on top of the social pressure to be a ‘good mother’ [11]. One participant described the effort required to continue with her PhD:

I almost quit a year or so ago…I was giving up every spare minute of my life to do this and it just seemed, my son wasn’t very well and other things, you just think why am I putting my energy here? What am I doing making myself miserable with this? But it has led to something great and exciting and exhausting (Female Interviewee 8, Midwife).

Another participant explained why she had decided to focus solely on clinical training after completing her Academic Clinical Fellowship, rather than progressing to do a PhD at this stage of her life:

I’ve got a young child…another on the way…I’ve also got my elderly grandmother nearby [and] my mother-in-law, and a lot of that falls on the female in the family, as much as husband’s around and very involved. And…I’m not sure I’ve got that in me actually, I don’t know if I’ve got a fifth gear…for the long view, I can only go up to fourth…You can’t drop the ball when it comes to home things, so you’re going to drop the ball in something at work; something’s got to give hasn’t it? (Female Interviewee 1, Academic Clinical Fellow)

This participant employed metaphors to describe her lack of sufficient energy to drive her career forward and also needing to carefully juggle various responsibilities, which she perceived as unsustainable long-term. Her comments also reveal the normative expectations for women to take responsibility for caring for the young and elderly in the family [11].

### Working part-time

The NMAHPs survey revealed that 34.3% of participants were employed on part-time contracts. However, this was not necessarily related to caring responsibilities but was likely to be for financial reasons, to supplement their funding. For example:

I’m registered for a full-time PhD and managed to negotiate my work: three days release for a PhD. But full-time means that something has to give; because trying to fit three days into a full-time PhD is impossible. So, it’s then evenings and weekends which impinge on family life (Male Interviewee 16 Allied Health Professional).

MCAs who worked part-time had different concerns:

It’s slowed down my training, if I was full-time I may well have been doing a clinical lectureship now and be almost a consultant…I’d be a lot closer to being able to apply for proper university posts (Female Interviewee 16, Academic Clinical Fellow).

As well as taking longer to qualify than their colleagues who pursue purely clinical training [4], working part-time entailed further delays. Another issue that arose amongst participants working part-time, was uneven splits between academic time and clinical duties. One participant reflected on the ‘never-ending’ nature of academic work, whilst another described being expected to cover a full-time clinical load in 3 days per week. Like many participants, she found it difficult to fit in the extra work needed for fulfilling promotion criteria and described writing papers and preparing grant applications after her children had gone to bed. These accounts reveal the additional challenges faced by clinical academics with caring responsibilities when trying to progress their careers, which is particularly problematic for those working part-time, the majority of whom are women. In addition, the issue of having no clear boundaries between clinical, academic, and family time highlights the need for contracts which clearly show how clinical academics should divide their time [2].

### Career progression

There were other ways in which working part-time was perceived as being detrimental to career development such as one survey respondent’s fear that it would deter potential funders.

Another area of potential disadvantage for clinical academics with young children was difficulties in attending conferences:

Getting to conferences has been a real challenge for me…I can’t go without taking [child] and that’s not very practical…The childcare finishes before the poster sessions [which end at 9.30pm]. If I was presenting a poster would I take him with me at bedtime and let him have a meltdown in the middle of the presentation? So how do I get the experience of presenting and networking and getting my research noticed? (Female Interviewee 15, Clinical Lecturer).

The above participant described steps taken by her university to enable female academics to attend conferences:

My institution is keen to help. They provide funding either towards additional childcare, or towards the costs of somebody travelling with you…But my husband doesn’t have the annual leave to follow me around the world doing the childcare, as supportive as he is and I often can’t get any extra days at [child’s] normal nursery, because they’re full. So I can’t use the funding (Female Interviewee 15, Clinical Lecturer).

This suggests that the impetus should be on conference organisers to be more child-friendly. Notably only one participant described a conference this way:

Last conference I went to there were lots of mums [with their] babies, and I was quite surprised, but it was brilliant to see how supportive it was. They had an area for breastfeeding, an area for relaxation…it was really good…With conferences and meetings, people bringing their babies means that they can get involved. If I had a child, I wouldn't hesitate in thinking about taking them with me (Female Interviewee 13, Clinical Lecturer).

The conference described above was for general practitioners, a female-dominated medical specialty [18]. However it reveals a concerning trend where in many cases, clinical academics with caring responsibilities are missing opportunities for dissemination, networking, and identifying potential collaborations, all of which are critical for career progression and research impact.

### Partner occupations

The topic of partners was raised in interviews with both NMAHPs and MCAs, many of whom described having partners in the same profession. Although some participants felt this was beneficial because their partners understood the pressures of the job and were able to share childcare by working opposite shifts, this risked them being ‘two ships passing in the night’ (Male Interviewee 7, PhD). Other issues were raised by participants without children who anticipated problems when both partners were ambitious:

We haven't adopted children [yet] and that's something we've discussed...but essentially neither of us wants to give up our career for that (Male Interviewee 9, Clinical Lecturer).

Both male and female participants said that having a partner with a ‘relatively normal’ job made things easier:

You tend to travel more as a clinical academic…being away for a month doing a research fellowship abroad or even a couple of weeks, that’s difficult. If you have young children especially. Luckily my wife is not in the same profession so that’s a bonus, I think. If your partner was another doctor, particularly at the same stage of training, that would have different demands. You have to have an understanding and supportive partner (Male Interviewee 14, Clinical Lecturer).

My luckiest point is that I have a husband who’s very supportive, he’s not a medic, or an academic, and so there is no competition in terms of who’s busier, and who’s more important or whatever. He’s just started working part time, so he’s quite flexible with the childcare, he does a lot of it (Female Interviewee 12, Clinical Lecturer).

These comments reveal that traditional views of women as primary caregivers were both perpetuated and challenged in the participants’ accounts.

### Childcare

In addition to partners contributing to childcare, participants described other arrangements that enabled them to combine clinical academic training with family responsibilities. For example, one participant who had no family living close by enlisted the help of her neighbours to provide emergency childcare.

Another described careful organisation of wider family members:

There's a lot of logistics….my husband starts late and he doesn’t have lunch…then he literally leaves work, picks [child] up takes him wherever he needs to go…my grandma’s, or his auntie’s…and then goes back to work till 8…We’re very lucky to have the family support around us, but I can't even begin to imagine what people do who don’t live near family (Female Interviewee 8, Academic Clinical Fellow).

This participant suggests that she relies on childcare from locally based family to enable her to continue her clinical academic training.

### Geographic limitations

An issue for participants who relied on local family members to provide childcare was the implication that they may not be able to consider moving location to progress their careers, as another participant described:

I have known people to give up clinical academic training due to family commitments which is a real shame. These ACF jobs are really hard to get…I know somebody who had to turn it down because it was too far away…She had kids and it wasn’t feasible; in the end she had to go for the normal clinical training. It’s really difficult…If there were more posts then it’s more feasible to have a family and work locally (Male Interviewee 11, Academic Clinical Fellow).

NMAHP participants raised other concerns, such as one who had relinquished her clinical post to pursue a PhD:

They said I’d have a job after [my PhD] but it could be anywhere in the Trust, so it’s not ideal…that’s the anxiety bit. I’ve got 3 children to consider (Female Interviewee 11, Allied Health Professional).

However, one participant suggested this was a gendered concern:

I feel like I'm thinking a lot more about the impact on my family with what I do than maybe some men would…I get the feeling that men are more willing to relocate their family…and I’m just not (Female Interviewee 10, Clinical Lecturer).

The above comments imply that women are more likely to consider their families when making career decisions, revealing gendered expectations of behaviour in relation to clinical academic careers.

### Gender stereotypes

Some participants’ experiences reflected societal expectations around women’s roles as caregivers. For example, one participant described being interviewed for a clinical lecturer post when she was pregnant:

I’d been encouraged to go for the job by someone saying, “They can’t discriminate against you”, but I was very aware that I needed to wear something that didn’t make me look pregnant…It was quite an intimidating interview, it was a panel of lots of middle-aged men, around a horseshoe table. I didn’t enjoy that interview at all…So I was surprised to then be offered the job. I suspect some people who had been part of that panel maybe felt slightly annoyed as well, [thinking] “Oh God, we’re going to have a woman who’s barely there” (Female Interviewee 12, Clinical Lecturer).

This participant’s pregnant body underlined her parental status, making her feel intimidated at being scrutinised by a homogenous panel of middle-aged men. Returning from maternity leave, the above participant reported a difficult start to her academic career:

I sent some emails to say hi to my Supervisor and I got some quite curt responses, and I’m guessing that it was because I’d been appointed and then I’d deferred for a year. I don’t know if that was frowned upon, but whatever, I was very anxious…I just remember thinking, ‘I feel like an imposter in medicine, an imposter in Academia’…I think starting was the hardest time, looking back (Female Interviewee 12, Clinical Lecturer).

These reported experiences suggest that this participant felt that she was perceived as less committed to a clinical academic career because she had taken time off to commit to raising her children, resulting in a loss of confidence.

### Interpersonal support

When asked what helped them to overcome challenges, the support of appropriate mentors was mentioned numerous times in participants’ survey and interview responses. When a suitable mentor was not provided, participants described making their own arrangements, such as through a scheme run by the Academy of Medical Sciences [19]. Another participant found support through social media:

There’s a Facebook group called, Physician Mums Group UK, but there’s a spin-off academic group. That’s actually good for asking questions and support and helping each other out, so that’s a whole group mentoring peer support thing (Female Interviewee 16, Academic Clinical Fellow).

These examples show how, in the absence of suitable mentors or peer support within their own institutions, participants found support through alternative means.

### Flexibility

Participants valued the flexibility of the clinical academic route which allowed them to work around family responsibilities and spend more time with their children:

There’s a lot more flexibility in an academic working day than there is an NHS working day. The universities don’t expect you to be there at a certain time, you’re just expected to do the results so if I need to do the school run on an academic day, that’s okay (Male Interviewee 14, Clinical Lecturer).

Flexibility was cited as an advantage, by another participant when discussing her plans:

During the second and third year where I'm going to be having 30-50% of the year as academic time…I see that as being a good time to have children. A lot of my colleagues have had children during their academic time because it does allow that little bit more flexibility with the working hours (Female Interviewee 3, Academic Clinical Fellow).

This comment reveals how some participants factored in their plans for parenthood within their career pathway, although conversely, one participant described people who have a baby while doing a PhD as ‘crazy!’

### Role models

As previously discussed, many participants spoke about being influenced by colleagues with children when planning their careers:

I intend to take maternity leave and also to work part-time at some point. I know this will likely be challenging but am encouraged from interactions with other clinical academics who have successfully done this (Female Survey Respondent 39, Clinical Lecturer).

However, the ability to do this seemed to depend upon the workplace environment:

The academic world is very male-dominated, and I think there is a certain degree of competitiveness and machismo…looking at the work/life balance that I see around, I think that it’s work, work, work. There aren’t many people who are a good example of getting that balance, which is what I need. That said, [another ACF] said it’s quite a different experience in her department. There are lots of females who have families who are able to manage that quite well (Female Interviewee 1, Academic Clinical Fellow).

These comments reveal the importance of having role models to show that it is possible to have a clinical academic career and a family. One participant described being inspired by her supervisor who remained available during two lots of maternity leave. Similarly, she continued with her research project during her own maternity leave and took her baby along to meetings.

Another participant described being perceived as a role-model:

A [female colleague] said, “look at all you’ve accomplished...you’re a clinical lecturer, you have a child, you have a family”. And I said, “you know what, until you said it, I don’t see having a child as an accomplishment.” I’m just like yeah, that’s par for the course (Female Interviewee 2, Clinical Lecturer).

This participant was uncomfortable with the implication that only women with ‘superhuman’ qualities can successfully combine a clinical academic career with family life. Her reaction suggests that for her, balancing family responsibilities is nothing unusual.

Other participants discussed ways in which they had developed time-management and organisational skills:

Having children makes you more focussed because your time is limited. If I need to get something done, I get something done…I can be very, very efficient which prior to having kids I wouldn’t be (Female Interviewee 16, Academic Clinical Fellow).

These experiences suggest that combining clinical academic training and family responsibilities actually enhances valuable skills that can enrich their professional profile, and that once in post, clinical academics with family responsibilities can bring numerous skills to the role.

## Discussion

This study of clinical academic trainees in the UK has revealed concerns about combining a clinical academic career with family responsibilities. Findings echo those of previous studies concerning work/life balance of academics generally [11], and medical academics specifically, the majority of which emanate from the USA [5, 6].

Unlike many studies which generally focus on women’s experiences of navigating work and family [11], the current study included both male and female participants. Nevertheless, there were some obstacles that only women reported [20]. Some of these reflected broader societal views of women’s roles in families [12, 21], resulting in some participants doubting their abilities to ‘have it all’. [11] Previous studies confirm that women with children are often perceived as being less committed to a clinical academic career [12], leading to suggestions that women’s progression may be hampered by stereotypical views that caring responsibilities are ‘women’s problems.’ [22]^,p.2038^ To a certain extent, this study’s findings perpetuate this notion. For example, male participants tended to discuss their families as part of their ‘breadwinner’ role, with financial security as their primary consideration when doing clinical academic training, even when they had small children. In contrast, many female participants discussed delaying training, or stepping off the clinical academic pathway, until their children were of school-age, seemingly prioritising motherhood over professional ambitions [11]. This also reflected childcare considerations which were mentioned more frequently by women in this study. In echoes of an American study from 20 years ago, male participants usually described their wives as providing most of the childcare [23], whereas female participants discussed networks of carers they relied upon, including not only their partners, but wider family members and neighbours. In these cases, childcare considerations not only impacted on the timing of their clinical academic training but also potentially restricted them geographically when seeking opportunities to progress their careers. This finding supports previous research which highlights a lack of mobility due to family commitments as a barrier to career progression for early-career clinical academics [24], and reinforces recommendations for an increase in the number and variety of posts available [24], (e.g. by introducing a research element to consultant posts) and/or relaxing eligibility so that people might return to the clinical academic pathway when their family circumstances change [8]. Such steps would avoid people (especially women) having to choose between keeping families together or prioritising their careers [1]. This is particularly important where both partners are ambitious and/or when relying on family support systems.

This study also revealed changes in family dynamics where, in many cases, male partners were contributing equally, if not more to childcare, enabling women to pursue their careers in ways traditionally associated with male clinical academics [12]. This however seemed to depend to a large extent on partners’ occupations. In line with previous research [12], both male and female participants considered themselves fortunate if their partner did not work in the medical profession and had regular working hours, enabling caring responsibilities to be more easily accommodated.

In addition to support at home, findings resonate with previous research emphasising the importance of effective mentors and role models championing balancing work with family responsibilities, particularly for women who work in male-dominated environments [5, 12, 24]. Although a shortage of female clinical academics may make it problematic to find suitable mentors and role models within particular institutions [1, 5, 20], participants found ways to source support through professional organisations and social media. This suggests that alternative sources of support should be publicised more widely in order to increase awareness of help which is available, including cross-institutional mentorship [20]. In addition, findings support recommendations that women in leadership roles should be more visible both in the clinical academic community, and in wider society, to demonstrate that it is possible to have both a successful career and a family [21].

This study has revealed difficulties for people with caring responsibilities to meet promotion criteria which emphasise publications and conference presentations [20]. Studies reveal gender disparities across academic disciplines, with female academics producing fewer publications than their male colleagues early in their careers (coinciding with the time when they are most likely to be childbearing or childrearing) [23]. This issue has been exacerbated during the Covid-19 pandemic where women have provided most of the childcare [25] and fewer manuscripts have been submitted by female academics [26], particularly early career women [27].As regards conferences, findings highlight a need to ensure that people with children are properly accommodated; allowing them opportunities to contribute to academic debates and to develop professional networks.

The findings also reveal specific talents, skills and insights that clinical academics with families can contribute to academia and the healthcare system, (e.g. strong organisational and management skills), supporting recommendations that these strengths should be recognised alongside traditional promotion criteria [21].

### Strengths and limitations of the study

In common with most qualitative research [13], this study is limited by the relatively small numbers of participants; a sample size which relied on the judgment of the researchers to establish when data saturation had been achieved [13]. However, in contrast to previous studies [1, 5, 7, 8], the participants came from a variety of medical professions and were at various stages of clinical academic training which enabled comparisons to be made across the data set. Perspectives were gathered from participants with young children, who were already juggling family responsibilities with clinical academic training, as well as participants who were yet to start a family, who were considering the career implications of doing so. This is important as perceived difficulties in continuing clinical academic careers alongside family responsibilities may lead to attrition of talented individuals, whereas steps can be taken to ensure that they feel supported to continue along the clinical academic pathway.

### Future research

The perspectives reported here are from clinical academic trainees, many of whom were yet to become parents. A follow-up longitudinal study, five or ten years hence, could explore how their careers have progressed. In addition, further research, currently underway, will explore ways in which female associate professors and (full) professors have managed to gain and sustain a successful clinical academic career alongside family caring responsibilities. In addition, it would be useful to compare clinical academics’ experiences of combining careers and family life with those of professionals who have pursued either a purely academic, or clinical, career.

## Conclusion

Higher education institutions, healthcare organisations and the National Institute for Health Research are committed to promoting equality, diversity and inclusion [15, 28, 29, 30]. However, there needs to be more consideration of how clinical academic trainees with family responsibilities can be supported to achieve their potential and avoid attrition of talented individuals. In addition, the value of their specific contributions to the clinical academic community, to healthcare and academic organisations, and ultimately to patient care, should be recognised and encouraged.

## Data Availability

The datasets generated and analysed during the current study are not publicly available due to identifiable data contained therein but are available from the corresponding author on reasonable request.

## Abbreviations

ACF: Academic Clinical Fellow(ship)
MCA: Medical Clinical Academic
NHS: National Health Service
NMAHPs: Nurses, Midwives and Allied Health Professionals
